# Impact of flagellar filament length on *Campylobacter jejuni* for colonization and flagellar-dependent phenotypes

**DOI:** 10.1128/jb.00199-25

**Published:** 2025-08-11

**Authors:** Alexis A. Waller, Deborah A. Ribardo, David R. Hendrixson

**Affiliations:** 1 Department of Microbiology, University of Texas Southwestern Medical Center 12334, Dallas, Texas, USA; University of Virginia School of Medicine, Charlottesville, Virginia, USA

**Keywords:** flagellar motility, FlhA, FlaG, flagellar filament, *Campylobacter jejuni*

## Abstract

Many bacteria produce flagella to swim and navigate through environments. Depending on the species, flagella are beneficial for other activities such as sensing surfaces to initiate biofilm development, secretion of specific proteins, and interactions with hosts to promote colonization or pathogenesis of disease. The extracellular region of the flagellum consists mostly of the filament, which is required for many flagellar-dependent activities. The length of the filament varies across bacterial flagellates and even stochastically within a clonal population, but it is unknown whether or how altering filament length impacts flagellar function for motility and other activities. We recently discovered a mechanism by which FlaG of *Campylobacter jejuni* and other polarly flagellated bacteria controls flagellar filament length so that these species produce shorter filaments than their peritrichous counterparts. We exploited a set of isogenic *C. jejuni* mutants producing significantly longer and shorter flagellar filaments than WT to assess the impact of filament length on activities of *C. jejuni* necessary for commensal colonization of a natural host. We found that production of shorter flagellar filaments slows swimming velocity and hinders intestinal colonization of chickens. Although production of shorter and longer flagellar filaments differently impacted cellular autoagglutination, only a subtle impact on biofilm formation was observed. We also acquired evidence that FlaG itself may impact biofilm development, suggesting that FlaG may have another function besides controlling flagellar filament length. Overall, our data suggest that shortening flagellar filaments has a greater impact on flagellar-dependent activities of *C. jejuni* than producing overly elongated filaments.

**IMPORTANCE:**

Flagella and flagellar motility are important to propel many bacteria through environments, biofilm formation, and infection of respective hosts. Most often, the importance of flagella for a species has been assessed with mutants lacking flagella altogether or only the extracellular filament while retaining the basal body. We addressed whether altering filament length impacts flagellar-dependent activities for host colonization. We exploited isogenic *Campylobacter jejuni* mutants that synthesize longer and shorter filaments on average relative to WT to find that production of shorter filaments had greater negative impacts on motility, autoagglutination, and commensal colonization compared to bacteria that produce elongated filaments. Our findings suggest that polar flagellates may produce a flagellar filament of minimal length to achieve flagellar functions and preserve fitness.

## INTRODUCTION

The bacterial flagellum is a nanomachine that functions as a reversible rotating propeller for swimming motility. This primary function of the flagellum is essential for bacteria to migrate and thrive in various environments. Many bacterial pathogens require flagellar motility for colonization and infection of hosts. For *Campylobacter jejuni*, flagella and flagellar motility are required by the bacterium to efficiently colonize and persist as a commensal in the intestinal tract of wild and agriculturally important animals, such as chickens, without causing disease (1–6). The *C. jejuni* flagellum is also essential for the bacterium to infect humans to cause diarrheal disease (7). As such, *C. jejuni* is the leading cause of bacterial gastroenteritis in humans in the United States and many other countries (8).

The flagellum is also involved in other functions for a bacterium that likely contribute to the infection of hosts. For *C. jejuni*, the flagella also promote autoagglutination and adherence to and invasion of host cells (9–13). Flagellar-mediated surface sensing is important in some bacteria for swarming motility, attachment to surfaces, and microcolony formation, which are initial steps in biofilm formation (14–16). In addition to secreting flagellar proteins to form the flagellar structure for swimming motility, the *C. jejuni* flagellum also secretes proteins involved in host association. These proteins include several Cia and Fed proteins and FlaC (17–25). Some of these proteins are required for association with and invasion of human intestinal cells, while others impact the ability of *C. jejuni* to promote colonization of chickens. However, the specific roles of many of these proteins are unclear.

*C. jejuni* is an amphitrichous flagellate that produces a single flagellum at both poles of the cell. *C. jejuni* synthesizes one of the most complex and largest flagellar motors in bacteria, characterized by more than a dozen additional proteins that contribute to a periplasmic scaffolding structure and an enlarged rotor to stably incorporate an increased number of stator units that drive flagellar rotation (26, 27). These features alter the mechanics of the *C. jejuni* flagellar motor to generate increased torque for high swimming velocities. As such, we observed that *C. jejuni* actually increases swimming velocity as viscosity increases to facilitate swimming speeds that are higher than many other bacterial flagellates (28). To swim in one direction, the opposing polar flagella simultaneously turn with the leading flagellum wrapped around the cell body and the lagging flagellum unwrapped (29). To change directions, a large basal disk structure functions as a flange to allow the leading flagellum to break contacts with the cell surface and unwrap to become the lagging flagellum, while the previously unwrapped lagging flagellum wraps around the cell body as the new leading flagellum (11, 29). During this process, both flagella switch rotational direction, which allows the bacterium to move along a different course. How the *C. jejuni* chemosensory system works on both flagellar motors at opposite ends of the cell to coordinate their rotation is not entirely known.

There are four major components of the flagellum: the flagellar type III secretion system (fT3SS) containing the export gate, rod, hook, and filament (30, 31). The fT3SS secretes most flagellar proteins that form the hollow rod and hook structure (30, 32, 33). At the tip of the hook is the flagellar filament that extends from 3 to over 10 µm from the cell surface and is composed of over 10,000 flagellins (34). Flagellins are delivered to the fT3SS export gate in the cytoplasm, secreted by the fT3SS into the channel within the rod to diffuse to the hook for incorporation at the tip of the filament (35–38). Stator units in the inner membrane associate with the rotor component of the fT3SS to power rotation of the flagellum for motility (39–41).

We and others have reported that polarly flagellated bacteria such as *C. jejuni* and many *Vibrio* and *Pseudomonas* species tend to produce shorter flagellar filaments than peritrichous flagellates like *Escherichia coli* and *Salmonella* species (42–46). We recently discovered a mechanism in *C. jejuni* for how the FlaG protein, which is conserved in polarly flagellated bacteria, limits the length of the flagellar filament so that these bacteria produce shorter filaments than their peritrichous counterparts (42). We found that FliS, which shuttles flagellins to the fT3SS export gate, and FlaG antagonize each other for binding to nearby docking sites on FlhA in the fT3SS export gate. FlaG association with FlhA lowers the ability of FliS to deliver flagellins to the fT3SS for secretion and filament polymerization. We also found that FlaG is secreted by *C. jejuni* into culture supernatants, presumably due to its association with FlhA (42). Although secretion of FlaG may not directly have a role in the regulation of flagellar filament length, it is unclear whether secretion of FlaG impacts another activity that benefits *C. jejuni*.

In many bacterial flagellates, the most common strategy to assess the importance of the flagellum for a certain phenotype is to compare WT and mutant strains that either completely lack flagellar biogenesis, eliminate production of the flagellar filament by mutation of genes encoding flagellins, or inactivate stators to result in a flagellum that does not rotate for swimming motility. To our knowledge, there is a general lack of studies that probe whether altering the length of the flagellar filament of bacteria impacts flagellar-dependent processes important for host interactions and colonization. In pursuit of understanding how FlaG impacts flagellar filament length via FlhA, we created a collection of isogenic *C. jejuni* mutants that lacked FlaG or contained point mutations in FlhA that resulted in flagellar filaments significantly shorter or longer than those produced by WT *C. jejuni* (42). In this study, we exploited this panel of isogenic mutants to investigate whether altering the length of the flagellar filament impacts the function of the flagellum for activities of *C. jejuni* required for infection, such as the swimming velocity, autoagglutination, biofilm formation, and secretion of virulence and colonization factors. Furthermore, we analyzed whether flagellar filament length impacts the ability of *C. jejuni* to colonize its natural avian host. Our results suggest that shortening flagellar filaments had a negative impact on flagellar motility and colonization in comparison to *C. jejuni* producing WT length filaments. In contrast, increasing flagellar filament length augmented aggregation of cells but did not impair colonization of chickens or swimming motility. Overall, our work provides insight regarding how flagellar filament length impacts certain activities of the bacterium that contribute to the infection of hosts.

## RESULTS

### Shortening flagellar filaments impacts the motility of *C. jejuni*

For this study, we used a subset of previously created isogenic *C. jejuni flaG* and *flhA* mutants that vary in the length of the flagellar filament they produce (Table 1 [42]). *C. jejuni* △*flaG* that lacks FlaG produces on average 77% longer flagellar filaments than WT *C. jejuni* (5.30 vs 3.03 µm; Table 1 [42, 43]). We also previously identified mutations in two regions of *flhA* that caused different filament length phenotypes. In *C. jejuni flhA*_F485A V513G_, the docking site for FliS-chaperone complexes on FlhA is altered so that flagellins are not efficiently delivered to the fT3SS for secretion. As a result, this mutant produces shorter flagellar filaments than WT (1.87 vs 3.03 µm; Table 1) with an increased level of FlaG secreted and a modest retention of flagellins in the cytoplasm (42). In *C. jejuni flhA*_N436A L439A_, we observed that FlaG interactions with FlhA were impaired, allowing FliS to deliver more flagellins to the fT3SS to cause production of longer flagellar filaments than WT (4.90 vs 3.03 µm; Table 1). In this mutant, FlaG accumulated in the cytoplasm due to reduced ability to interact with FlhA for secretion (42).

**TABLE 1:** Filament lengths of *C. jejuni* mutants analyzed in this study

	FlaG	Mean filament
Strain^*a*^	Produced	Length (µm ± SD)
WT	Yes	3.03 ± 0.66
△*flaG*	No	5.30 ± 2.30
*flhA*_F485A V513G_	Yes	1.87 ± 0.37
*flhA*_N436A L439A_	Yes	4.90 ± 1.67
△*flaG flhA*_N436A L439A_	No	5.18 ± 2.17

^*a*^ All strains and lengths were reported in Waller et al. (42).

**Fig 1:**
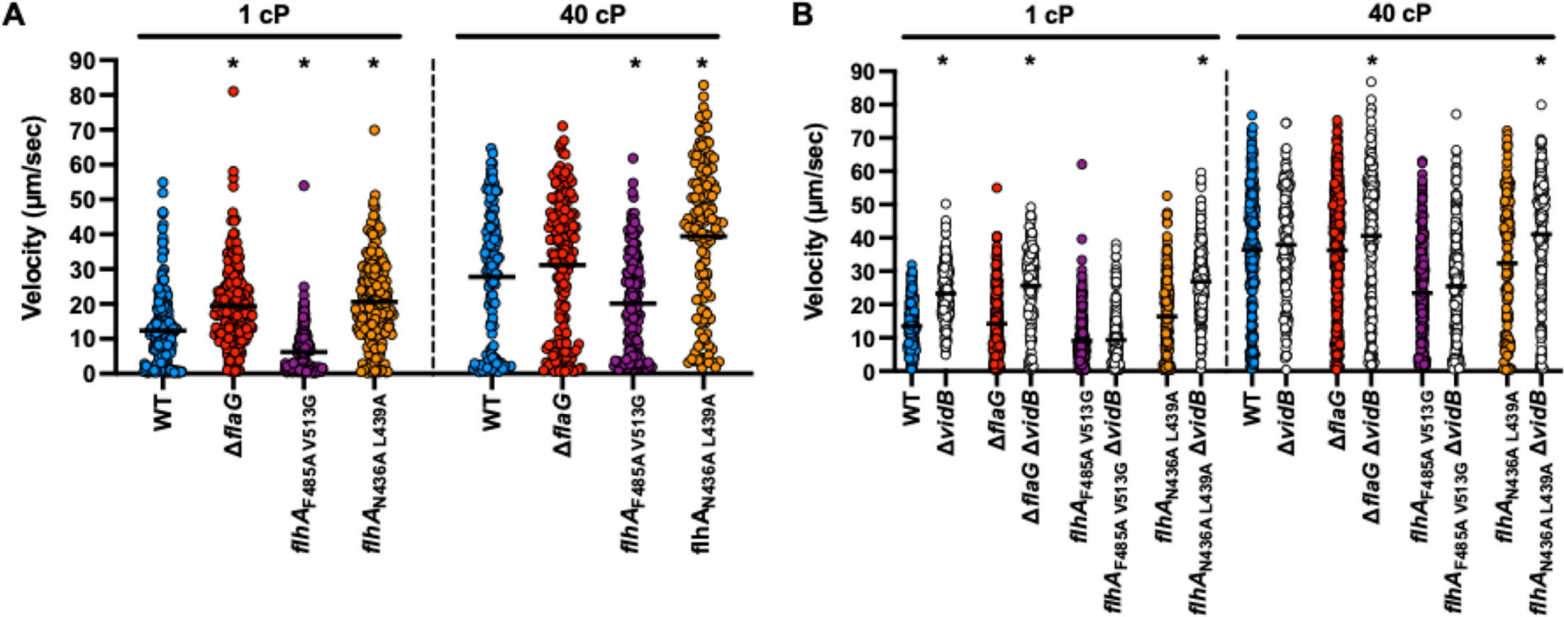
Swimming velocities of WT *C. jejuni* and flagellar filament length mutants in media with different viscosities. (**A and B**) Swimming velocity of WT *C. jejuni* and mutant populations producing different filament lengths after growth for 24 h in Mueller Hinton (MH) broth alone (1 cP) or with methylcellulose to increase viscosity to 40 cP. Swimming velocities of individual cells (*n* > 100) were measured by video tracking under dark-field microscopy. Circles represent individual cells. The bar represents the mean swimming velocity of the population. For panel **A**, statistical significance between WT and mutants at 1 cP or 40 cP was calculated by one-way analysis of variance (ANOVA) followed by Tukey’s multiple comparisons test (*P < 0.05). In panel **B**, *vidB* mutants of each strain are shown as white circles. Statistical significance of each strain to its *vidB* isogenic mutant was calculated by ANOVA followed by Tukey’s multiple comparisons test (*P < 0.05).

We first assessed the impact of filament length on flagellar motility by measuring the swimming velocity of individual cells in liquid media of two different viscosities. We previously observed that *C. jejuni* swimming velocity increased as the viscosity of the environment increased (28), presumably due to the evolution of *C. jejuni* to maintain residence and persist in the viscous mucus layer atop the lower intestinal tract of avian and animal hosts and infect the human intestinal tract to cause diarrheal disease (6, 47, 48). For this analysis, we grew WT and mutant *C. jejuni* strains in microaerobic conditions at 37°C in Mueller-Hinton broth alone (1 cP viscosity) or with the branched-chain polymer methylcellulose at a concentration to achieve 40 cP (approximately the viscosity of corn oil at ambient temperature). The motility tracks of over 100 individual cells were recorded by dark-field microscopy and then converted to swimming velocities. As reported previously, the mean swimming velocity of individual WT *C. jejuni* cells was 12.4 µm/s at 1 cP but increased to 27.8 µm/s in 40 cP broth (Fig. 1A). For *C. jejuni* △*flaG* that produced longer flagellar filaments on average, we observed an increase in mean swimming velocity to 19.4 µm/s at 1 cP, which was statistically significant. These cells also showed an enhanced swimming velocity in media at 40 cP relative to 1 cP, but the velocity at 40 cP was similar to WT. Consistent with our findings for △*flaG*, the mean swimming velocity of *C. jejuni flhA*_N436A L439A_ cells that also produced longer flagellar filaments was higher than WT at 1 cP (20.6 µm/s) and 40 cP (39.4 µm/s; Fig. 1A). In contrast, *C. jejuni flhA*_F485A V513G_ that produced shorter filaments than WT *C. jejuni* demonstrated an average swimming velocity significantly lower than WT at both 1 cP (6.2 vs 12.4 µm/s) and 40 cP (20.2 vs 27.8 µm/s; Fig. 1A). These results suggest that producing shorter flagellar filaments than the WT strain impairs swimming motility in both high- and low-viscosity environments.

Results from our previous study suggested that the *C. jejuni* flagellar motor, by default, is maximally powered to generate high torque for high swimming velocities in viscous environments (28). To lower swimming velocity in low-viscosity conditions, *C. jejuni* has evolved VidA and VidB to function together in a brake or clutch-like mechanism to impact motor activity. VidB represses motor output in low-viscosity conditions, and VidA counteracts VidB activity to allow for a modest, WT swimming velocity in low viscosity (28). In the absence of *vidA*, VidB has a dominant effect to greatly impede swimming velocity in media at 1 cP. In high-viscosity conditions, both VidA and viscosity itself completely eliminate the repressive effect of VidB to allow for the highest swimming velocities observed by *C. jejuni* (28).

We investigated whether the lower swimming velocity produced by *C. jejuni flhA*_F485A V513G_ cells was due to the short filaments unable to function optimally for motility or an inability of the flagellar motor to overcome the repressive effect of VidB at 1 cP. Deletion of *vidB* from WT *C. jejuni* and the △*flaG* and *flhA*_N436A L439A_ elongated filament mutants caused a statistically significant increase in swimming velocity at 1 cP (Fig. 1B), which is consistent with a reduced braking or clutch activity by removal of VidB. However, the swimming velocity of *flhA*_F485A V513G_ cells that produce short flagellar filaments did not change at 1 cP upon deletion of *vidB* (Fig. 1B). Analysis of strains and their *vidB* mutant counterparts at 40 cP revealed that all strains increased swimming velocity (Fig. 1B). Notably, *flhA*_F485A V513G_ cells with or without *vidB* continued to swim with similar lower velocities than WT *C. jejuni* regardless of the viscosity of the media, indicating that production of shorter flagellar filaments functionally hinders *C. jejuni* swimming motility even in a *vidB* mutant background with a fully defective braking mechanism (Fig. 1B).

### Flagellar filaments of different lengths differently affect autoagglutination

Flagellated *C. jejuni* strains autoagglutinate due to cell-cell interactions between glycosylated flagellar filaments and other surface structures like glycoproteins and capsular polysaccharide (10, 11, 49–51). The *C. jejuni* flagellar filament is composed of two flagellins, the FlaA major flagellin and the FlaB minor flagellin, that share 95% identity in amino acid sequence (52, 53). FlaB is secreted during assembly of the rod and hook and composes a short, proximal segment of the filament, whereas FlaA composes the majority and remainder of the filament (52). Each flagellin has up to 19 potential serine or threonine residues that are post-translationally modified with *O*-linked glycans such as pseudaminic acid, legionaminic acid, or specific derivatives (51, 54, 55). Five specific glycosylated residues are involved in mediating interactions with flagella and surface components of other *C. jejuni* cells, whereas glycosylation of three specific serine or threonine residues is required for packing of flagellins to construct the flagellar filament (10).

**Fig 2:**
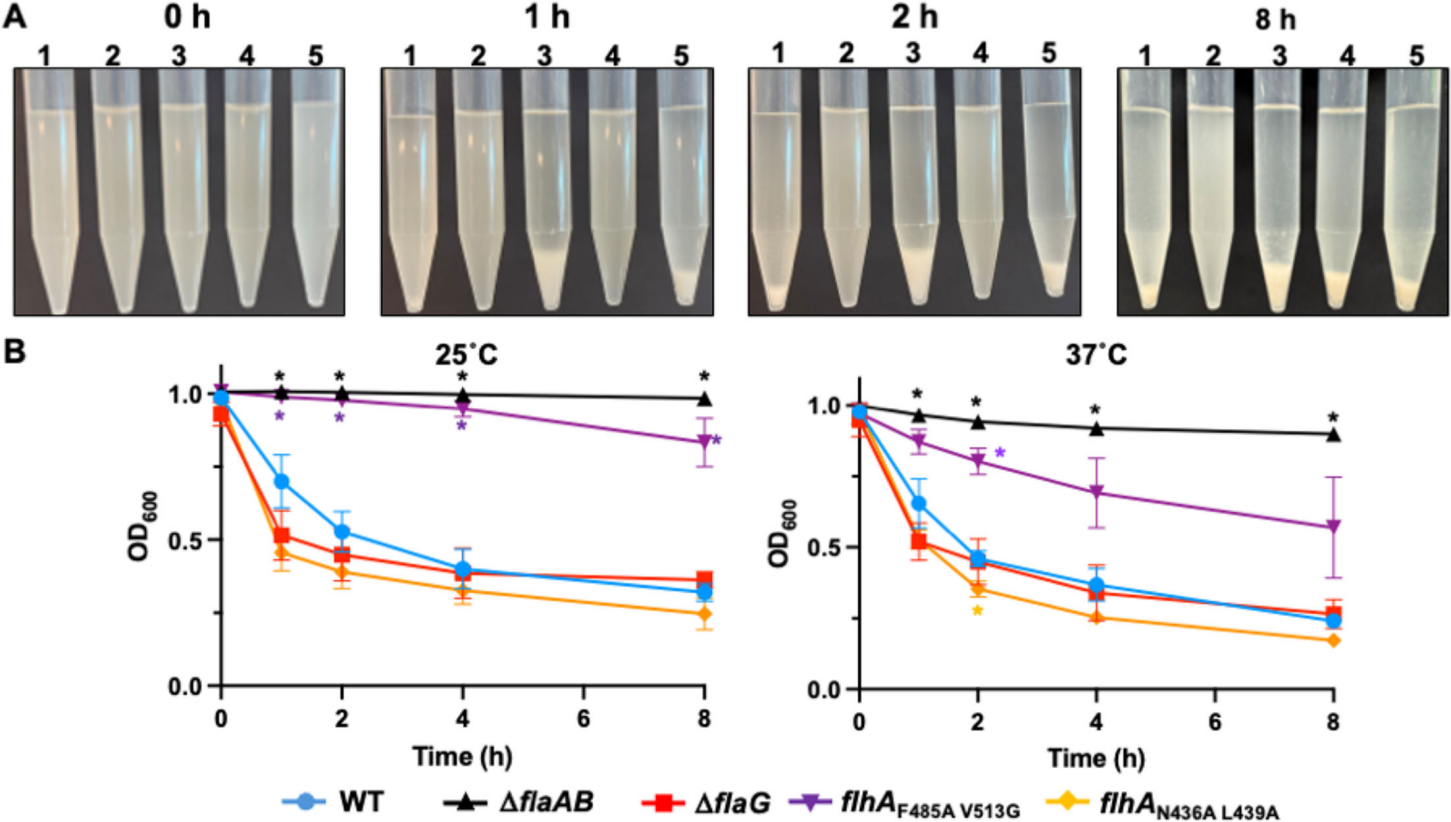
Autoagglutination of WT *C. jejuni* and flagellar filament length mutants over time. (**A**) Autoagglutination of *C. jejuni* cells in standing cultures. WT *C. jejuni* and mutant strains were diluted to equal densities in 10% MH broth-PBS and were allowed to stand stationary in conical tubes at 25°C for up to 8 h. Images of cultures were taken at 0, 1, 2, and 8 h. Strains include (1) WT *C. jejuni*; (2) △*flaAB*; (3) △*flaG*; (4) *flhA*_F485A V513G_; and (5) *flhA*_N436A L439A_. (**B**) Quantitative analysis of autoagglutination of *C. jejuni* strains in standing cultures. WT *C. jejuni* and mutant strains were diluted to equal densities in 10% MH broth-PBS and were allowed to stand in cuvettes at (left) 25°C or (right) 37°C for up to 8 h. OD_600_ readings of suspensions to monitor clearing due to cell aggregation were recorded over time. Each point indicates the average OD_600_ reading over three replicates. Error bars indicate the SD. Statistical significance between WT and mutants was calculated by two-way analysis of variance (ANOVA) followed by Dunnett’s multiple comparisons test (*P < 0.05).

We first analyzed whether the production of different flagellar filament lengths affects gross autoagglutination of WT *C. jejuni* and mutants in standing cultures. For this approach, overnight growth of *C. jejuni* cells was normalized to an OD_600_ of 1.0 in 10% Mueller Hinton (MH) broth-90% phosphate-buffered saline (PBS) solution, and the formation of a visible pellet in stationary conical tubes at 25°C was tracked over time (29). We observed WT *C. jejuni* forming a pellet at the bottom of the tube due to autoagglutination after 1 h, and the size of the pellet increased over time (Fig. 2A). This autoagglutination was dependent on the presence of a flagellar filament, as a similar pellet was not observed in the △*flaAB* mutant, which produces a flagellar basal body and hook structure but lacks an extracellular filament. *C. jejuni* △*flaG* formed a large pellet after just 1 h (Fig. 2A). However, this aggregate was more loosely packed than that of WT *C. jejuni*, suggesting that production of longer flagellar filaments may interfere with the architecture of *C. jejuni* aggregates. We observed a similar, faster-forming, yet loosely packed aggregate with *flhA*_N436A L439A_, to further support that elongated flagellar filaments enhance autoagglutination. In contrast, *C. jejuni flhA*_F485A V513G_, which produces shorter flagellar filaments than WT, was delayed in autoagglutination, but autoagglutination did occur by 8 h.

**Fig 3:**
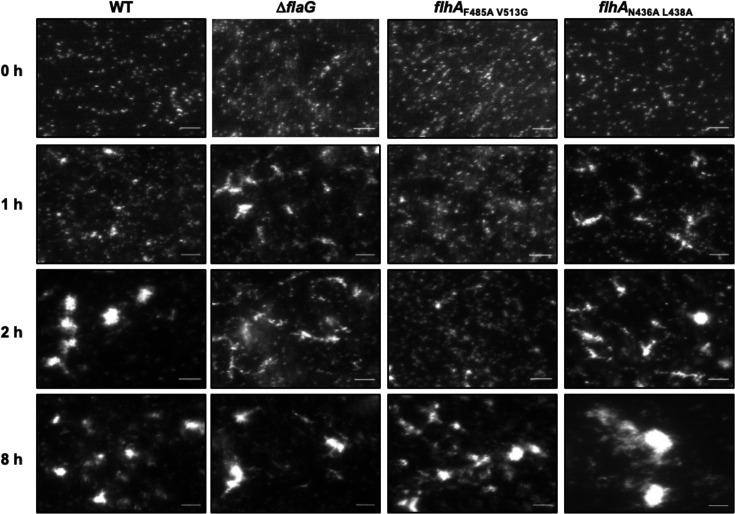
Formation of aggregates due to autoagglutination over time. Low magnification dark-field microscopy images of standing cultures of WT *C. jejuni* and flagellar filament length mutants at 25°C over an 8 h time course to visualize the formation and architecture of cellular aggregates during autoagglutination. Bar = 15 µm.

We next analyzed whether the aggregates formed by WT *C. jejuni* and long flagellar filament mutants during autoagglutination appeared visually distinct to possibly contribute to the different formation of the pellets in standing cultures. We repeated the autoagglutination assay with a glass slide inserted in stationary cultures in the conical tubes at 25°C and then visualized individual cells and aggregates by dark-field microscopy. At the beginning of the experiment (0 h), single cells were predominant in all cultures with little to no aggregate formation (Fig. 3). For WT *C. jejuni*, a few small, rather globular aggregates were observed at 1 h. These globular aggregates increased in size and number after 2 and 8 h (Fig. 3). *C. jejuni* △*flaG* and *flhA*_N436A L438A_, which both produce elongated flagellar filaments, formed more aggregates than WT after 1 h in standing cultures (Fig. 3). This observation is consistent with the earlier pellet formation observed in conical tubes (Fig. 2A). In contrast to WT globular aggregates, the aggregates formed by △*flaG* and *flhA*_N436A L438A_ were extended and more fibrous in architecture (Fig. 3). The greater surface area of these aggregates relative to the globular aggregates formed by WT may contribute to the less compact pellets observed in standing cultures of *C. jejuni flaG* and *flhA*_N436A L438A_ (Fig. 2A). These aggregates became more globular in shape like those of WT *C. jejuni* at 2 h with *flhA*_N436A L438A_ and at 8 h with △*flaG* (Fig. 3), which may have caused formation of the more compact pellet observed in conical tubes at these time points (Fig. 2A). For the *flhA*_F485A 513G_ mutant that produced short flagellar filaments, there was a general delay in formation of aggregates, but the aggregates appeared more globular like those of WT *C. jejuni* when they formed. This observation is consistent with the delay in standing cultures of this mutant forming a pellet (Fig. 2A). Overall, these data suggest that the formation of longer flagellar filaments by *C. jejuni* alters the shape and rate of aggregate formation.

Given the robust autoagglutination observed in elongated filament mutants, we pursued a more quantitative assessment of the impact of *C. jejuni* flagellar filament length on autoagglutination. Overnight cultures of strains were again normalized to an OD_600_ of 1.0 and then placed in standing cultures at 25°C or 37°C for up to 8 h. At designated time points, OD_600_ reading of the standing cultures was obtained to measure the amount of clearing of the culture as the cells autoagglutinated from the solution. In WT, a 30% decrease in OD_600_ was observed within 1 h at both temperatures, which continued to drop to 50% by 2 h and plateaued to a 63% decrease in density by 8 h (Fig. 2B). No more than a 2% decrease in OD_600_ was observed in the flagellinless △*flaAB* mutant over time, indicating that filament production was essential for autoagglutination. In contrast, the *C. jejuni* △*flaG* and *flhA*_N436A L439A_, which both produce elongated filaments, showed more rapid autoagglutination than WT *C. jejuni*, with a 45%–53% decrease in optical density observed after 1 h, confirming the rapid autoagglutination we observed in conical tubes by visual examination (Fig. 2AB). At later time points, only minor additional decreases in OD_600_ were observed. Consistent with some ability to autoagglutinate, *C. jejuni flhA*_F485A V513G_ showed an intermediate autoagglutination phenotype relative to WT *C. jejuni* and △*flaAB*, with 6%–17% decrease in OD_600_ from standing cultures after 4–8 h (Fig. 2B). These data support that altering flagellar filament length impacts *C. jejuni* autoagglutination, with shorter filaments hindered from autoagglutination and longer filament lengths facilitating more rapid autoagglutination with aggregates that are less compact relative to those of WT *C. jejuni*.

### FlaG is important for biofilm formation

**Fig 4:**
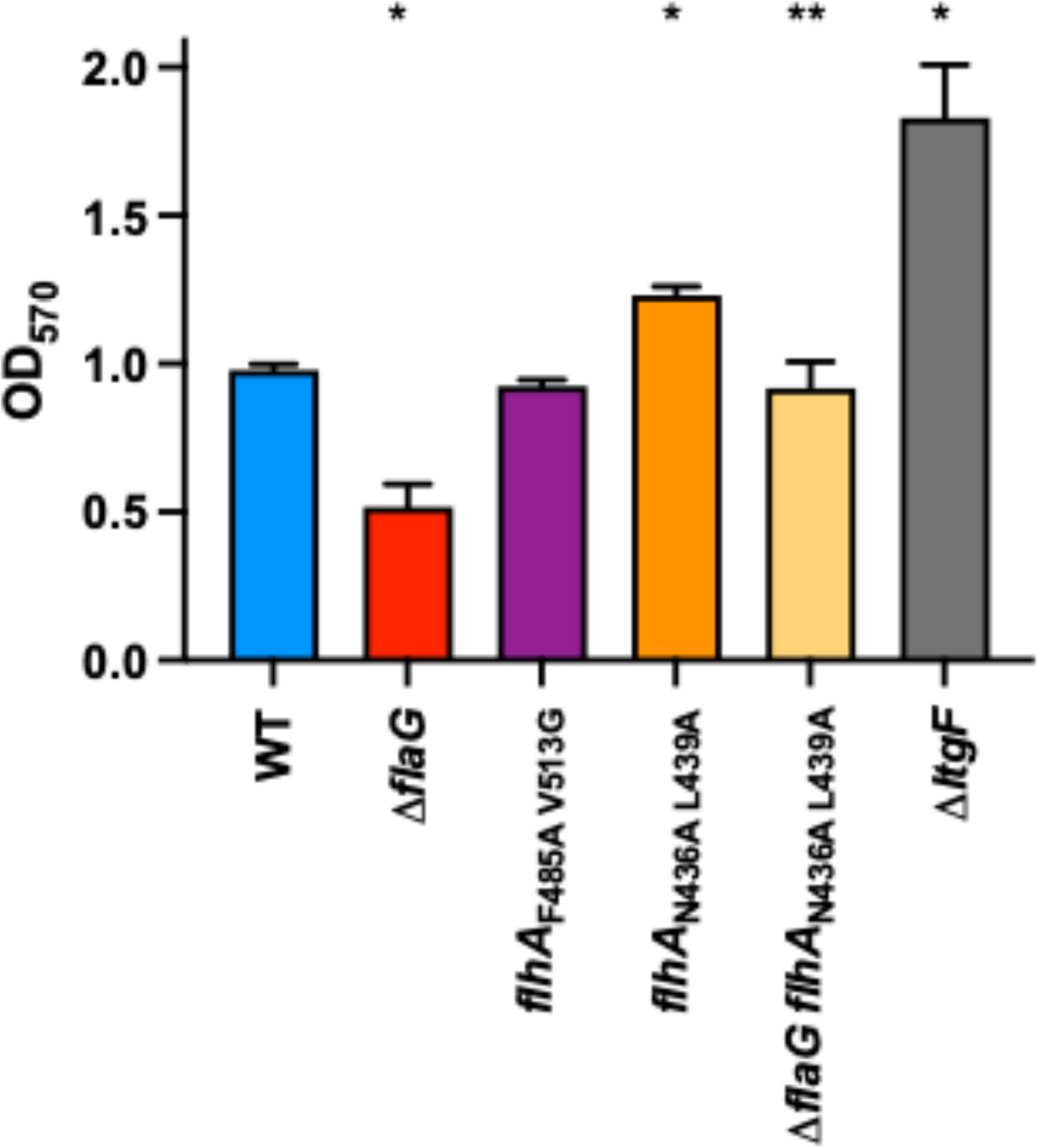
Biofilm formation of WT *C. jejuni* and flagellar filament length mutants. Quantification of crystal violet staining at OD_570_ of WT *C. jejuni* and isogenic △*flaG*, *flhA*_F485A V513G_, *flhA*_N436A L439A_, △*flaG flhA*_N436A L439A_, and △*lgtF* mutants. Error bars represent the SD from an assay with strains tested in triplicate. Statistical significance between strains was calculated by one-way analysis of variance (ANOVA) followed by Tukey’s multiple comparisons test (*P < 0.05, statistically significant differences between WT and mutants; **P < 0.05, statistically significant differences between *flhA*_N436A L439A_ and △*flaG flhA*_N436A L439A_).

Swimming motility and flagella are important for *C. jejuni* biofilm formation, with flagella assisting in mediating surface contact to initiate biofilms (56–58). To determine the impact of flagellar filament length on biofilm formation, we monitored biofilm formation of standing cultures in 24-well polystyrene plates after 3 d by crystal violet staining of biofilm mass. For WT *C. jejuni*, modest staining was observed with an average OD_570_ of 0.98 (Fig. 4). Previous studies have shown that a *C. jejuni* △*lgtF* mutant that alters the lipooligosaccharide outer core on the surface of the bacterium was enhanced for biofilm formation (59). We observed almost a doubling of biofilm formation for △*lgtF* relative to WT (Fig. 4), as shown previously (59). Production of shorter flagellar filaments by *C. jejuni flhA*_F485A V513G_ did not impair biofilm formation compared to WT *C. jejuni*. In contrast, *C. jejuni* △*flaG* that produced longer filaments than WT showed a 50% reduction in biofilm formation, suggesting that increasing flagellar filament length may hinder biofilm development. However, *C. jejuni flhA*_N436A L439A_, which also produces longer filaments due to mutation of the interaction site of FlaG on FlhA, actually showed a ~24% increase in biofilm mass, suggesting that production of longer flagellar filaments alone did not hinder biofilm formation. We considered whether FlaG itself may have a role in biofilm formation outside of its role in regulating flagellar filament length. We previously observed that removal of *flaG* from *C. jejuni flhA*_N436A L439A_ allowed the mutant to continue to produce elongated filaments. These average filament lengths between *flhA*_N436A L439A_ and △*flaG flhA*_N436A L439A_ were similar and both elongated compared to WT *C. jejuni* ([42]; Table 1). We observed that *C. jejuni* △*flaG flhA*_N436A L439A_ showed a 25% decrease in biofilm formation relative to its parent strain, suggesting that FlaG may directly impact biofilm formation outside of a role in regulating flagellar filament length.

### Flagellar-dependent secretion of proteins is not impacted by flagellar filament length

In addition to secreting FlaG and flagellar proteins that form parts of the flagellum, the *C. jejuni* flagellum also secretes factors necessary for colonization, such as the Cia and Fed proteins, which are not required for flagellar motility (18–25, 60). We have shown that two such proteins, FedB and CiaI, are both required for WT levels of colonization of the ceca of chicks, whereas CiaI has an additional role in assisting in invasion of human intestinal epithelial cells (19, 20, 23). We investigated whether altering flagellar filament length, especially lengths significantly longer than WT *C. jejuni*, might impair the *C. jejuni* flagellum in secreting proteins extracellularly. We recovered supernatant proteins from overnight cultures of WT *C. jejuni* and mutants with different flagellar filament lengths. We also employed a *C. jejuni* △*fliJ* mutant that is impaired for energizing the fT3SS for protein secretion as a negative control (20).

**Fig 5:**
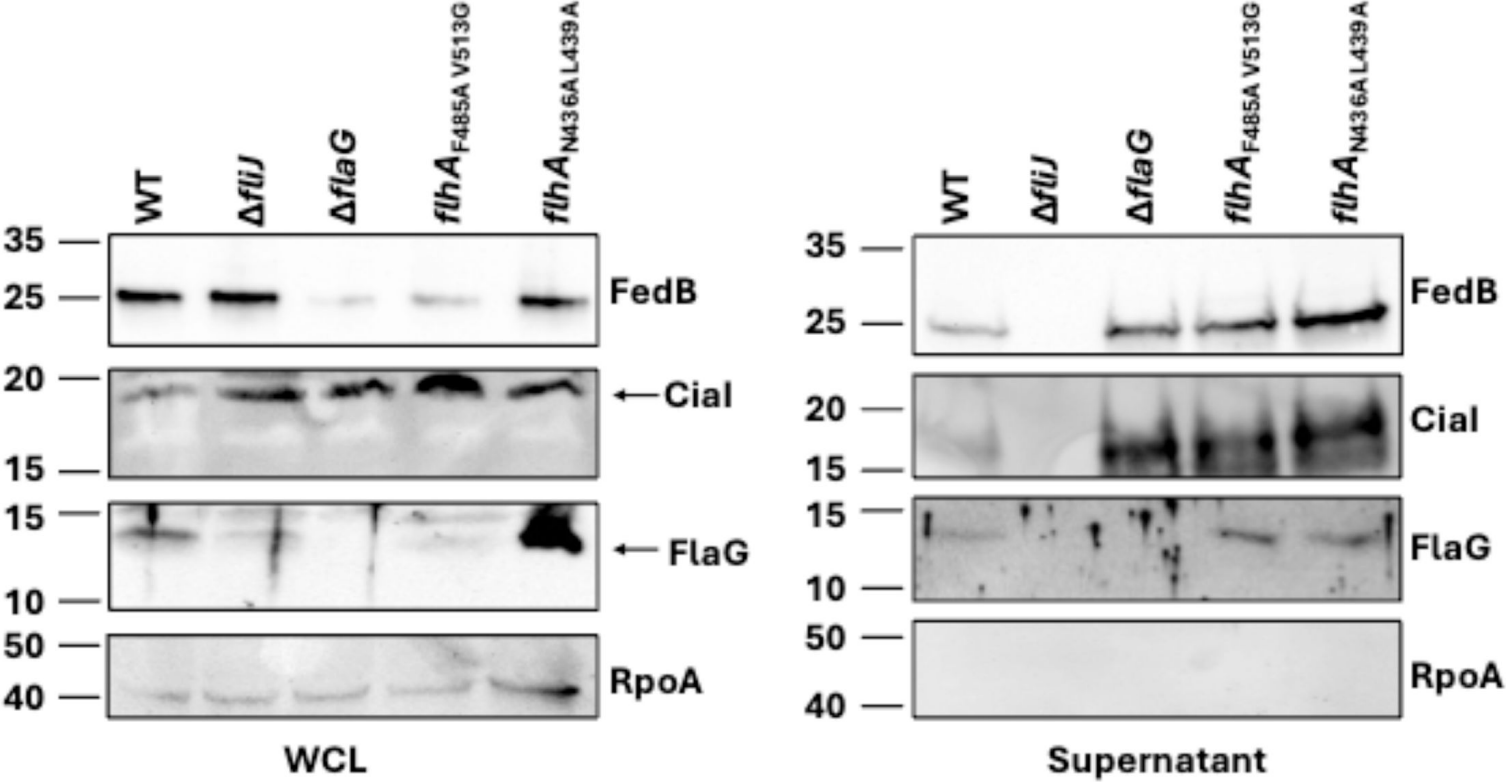
Analysis of flagellar-dependent protein secretion in WT *C. jejuni* and flagellar filament length mutants. Immunoblot analysis of FedB, CiaI, FlaG, and RpoA levels in whole-cell lysates (WCL) and supernatants of WT *C. jejuni* and isogenic mutants. Specific antiserum was used to detect FedB, CiaI, FlaG, and RpoA. Detection of FlaG served as a control for the recovery of secreted proteins. Detection of RpoA served as a control for equal loading of WCLs and the absence of cytoplasmic proteins in supernatant.

As reported previously, we observed WT *C. jejuni* to secrete FedB, CiaA, and FlaG into supernatants (Fig. 5 [19, 20, 42]). In contrast, these proteins were not secreted by the *C. jejuni* flagellum and fT3SS in the △*fliJ* mutant. Despite differences in flagellar filament lengths between *C. jejuni* △*flaG* and *flhA*_N436A L439A_, which produce elongated filaments, and *flhA*_F485A V513G_, which produces a short flagellar filament, levels of secretion of FedB or CiaI from the flagellum to the supernatants were similar. As we observed previously (42), cell-associated FlaG levels were increased in *flhA*_N436A L439A_ due to a lack of interaction with FlhA, although some FlaG was still secreted (Fig. 5; Fig. S1). Little FlaG remained in *C. jejuni flhA*_F485A V513G_ cells due to its increased secretion. As a control, we did not detect RpoA in culture supernatants, confirming that we were only analyzing secreted proteins and not cell-associated proteins in our preparations (Fig. 5; Fig. S1). Overall, our analysis suggests that flagellar filament length does not impact flagellar-dependent secretion of proteins from *C. jejuni*, at least to the maximal lengths synthesized by the *C. jejuni* △*flaG* and *flhA*_N436A L439A_ mutants.

### Altering flagellar filament length causes different effects on the commensal colonization capacity of *C. jejuni*

**Fig 6:**
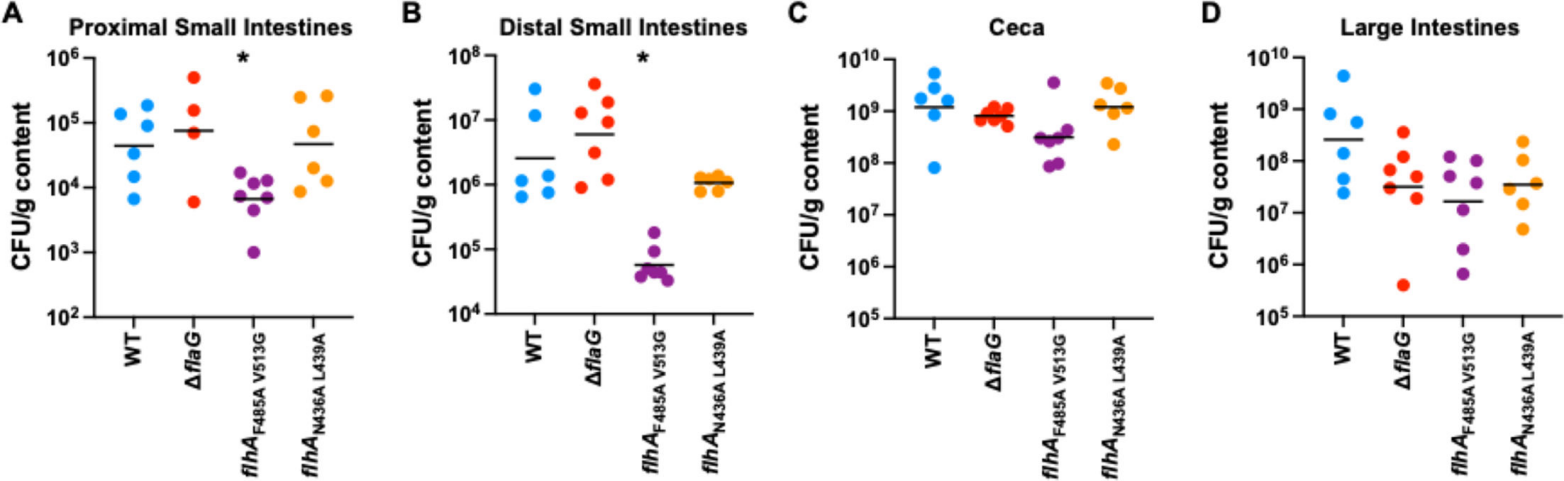
Colonization capacity of WT *C. jejuni* and flagellar filament length mutants for the chick intestinal tract. Day-of-hatch chicks were orally infected with ~100 cfu of each *C. jejuni* strain. Chicks were sacrificed at day 7 post-infection, and levels of each *C. jejuni* strain were determined in the (**A**) proximal small intestine, (**B**) distal small intestine, (**C**) ceca, and (**D**) large intestine and reported as the number of cfu per gram of organ content. Each circle represents the level of *C. jejuni* in the organ of a single chick. The bar represents the geometric mean. Statistical significance between loads of WT and mutants in each organ was calculated by Mann-Whitney test (*, *P* < 0.05).

To discern whether filament length impacts commensal colonization of a natural avian host, we examined the ability of WT *C. jejuni* and mutant strains to colonize throughout the intestinal tract of chicks. Day-of-hatch chicks were orally gavaged with ~100 cfu of each *C. jejuni* strain, and the load of *C. jejuni* in each intestinal organ was determined at day 7 post-infection. As we previously reported (2), WT *C. jejuni* colonizes throughout the intestinal tract with lower levels in the proximal and distal regions of the small intestines (4.4 × 10^4^ and 2.55 × 10^6^ cfu/g organ content, respectively) and higher levels in the ceca and large intestines (1.2 × 10^9^ and 2.59 × 10^8^, respectively; Fig. 6A through D). For the *C. jejuni* △*flaG* and *flhA*_N436A L439A_ mutants that produced elongated flagellar filaments, we did not observe defects in colonization throughout the small intestines or ceca (Fig. 6A through C). However, *C. jejuni flhA*_F485A V513G_, which produced shorter flagellar filaments than WT, showed significant 6.7- and 44-fold lower levels of colonization in the proximal and distal small intestines, respectively (Fig. 6AB). This mutant also was reduced 3.8-fold for colonization of the ceca compared to WT *C. jejuni* (Fig. 6C). In the large intestines, we consistently found that both types of flagellar filament length mutants—those that produce significantly longer and shorter flagellar filaments than WT—had 7.4- to 15.6-fold lower levels of colonization, although this difference did not reach statistical significance (Fig. 6D). These data suggest that production of shortened flagellar filaments is more detrimental for colonization of the avian intestinal tract, especially in the small intestines. Our data also suggest that alteration of flagellar filament length, either by producing longer or shorter filaments than WT, may reduce the ability of the bacterium to optimally colonize the large intestines of the host.

## DISCUSSION

The aim of this study was to understand how flagellar filament length impacts flagellar-dependent activities important for the lifestyle of a bacterium, with the opportunity to provide insight into why polar flagellates generally produce shorter flagellar filaments than their peritrichous counterparts. While the flagellum and its structure are known to be essential or important for a wide range of activities for diverse bacterial species, there have been insufficient studies to specifically examine whether the length of the flagellar filament impacts the flagellum for different processes. In this report, we took advantage of isogenic *C. jejuni* mutants that produce shorter or longer flagellar filaments relative to WT *C. jejuni* to address how filament length impacts flagellar-dependent activities, which avoided comparing WT and mutant strains that completely lack flagella or the filament altogether.

*C. jejuni* has evolved a high-torque flagellar motor to enable the bacterium to swim with high velocity through viscous conditions that normally impede or reduce swimming of many other bacterial flagellates (26, 28, 61–63). We determined that reducing filament length, as seen in *C. jejuni flhA*_F485A V513G_, caused decreased swimming velocity at 1 cP (the viscosity of water). However, at 40 cP (roughly the viscosity of corn oil), this defect was less pronounced relative to WT *C. jejuni*. Elimination of VidB, which functions as a brake or clutch to lower swimming velocity (28), did not increase the speed of swimming of *C. jejuni flhA*_F485A V513G_. These data indicate that shortening the flagellar filament impacts the ability of the flagellum to effectively propel the bacterium, rather than impacting the external load on the filament to alter the mechanics of stators, rotor, and/or VidB to efficiently turn the flagellum.

We observed that *C. jejuni* △*flaG* and *flhA*_N436A L439A_, which produced elongated flagellar filament lengths, swam modestly faster than WT *C. jejuni* at either 1 or 40 cP at least in our *in vitro* conditions. We predicted that elongated filament mutants may display reduced swimming velocity because the longer filaments could require more force to rotate the flagellum, given a presumed increase in external load on the extended flagellar filament due to viscous drag. However, this was not the case. It is possible that elongated flagellar filaments could impair migration in the mucus layer lining the intestinal epithelium of the human or avian host, which has a range of viscosities. However, *C. jejuni* mutants producing elongated filaments were not impaired for short-term colonization in the small intestines or ceca.

Autoagglutination of *C. jejuni* cells is known to occur by glycosylated epitopes that modify flagellin subunits and aid in polymerization of the flagellins into the filament (10). We observed that the *C. jejuni* mutants like △*flaG* and *flhA*_N436A L439A_ with elongated flagellar filaments consistently autoagglutinated more rapidly than WT *C. jejuni*, presumably due to more glycosylated flagellin subunits per each elongated filament on the bacterial cells. The *C. jejuni* short filament mutant autoagglutinated less, which further supported that the number of flagellins in the filament correlates with the rate of *C. jejuni* autoagglutination. We also found that the aggregates formed by the *C. jejuni* elongated flagellar filament mutants during autoagglutination were visually distinct from those formed by WT *C. jejuni*. These aggregates were initially more fibrous or thread-like in form but became more globular like WT over time. The compact globular aggregate made by WT *C. jejuni* cells may be due to more direct contacts between neighboring cell bodies and filaments. The extended flagellar filaments of the *C. jejuni* △*flaG* and *flhA*_N436A L439A_ mutant may partially impede close cellular contacts and delay formation of globular aggregates.

We anticipated that differences in flagellar filament length and autoagglutination may affect the ability of the *C. jejuni* mutants to form biofilms. However, we did not find a correlation between temporal aspects of autoagglutination and the degree of biofilm formation. Unlike in other species such as *Pseudomonas aeruginosa* or *Vibrio cholerae*, where many factors contribute to genetic and physiological programming for biofilm development, much less is known in *C. jejuni* regarding requirements for biofilm formation. In addition, *C. jejuni* lacks type IV pili and cyclic di-GMP signaling that are major factors in biofilm formation in other species. Although *C. jejuni* △*flaG* and *flhA*_N436A L439A_ are phenotypically similar in terms of flagellar filament length and more rapid cellular autoagglutination, one difference is that FlaG is absent from △*flaG*, but FlaG cytoplasmic levels are greatly increased in *flhA*_N436A L439A_, with some secreted extracellularly from the cell. Thus, it is possible that FlaG itself is involved in biofilm development and has another role in *C. jejuni* besides regulating flagellar filament length. As such, elimination of *flaG* from *flhA*_N436A L439A_ did reduce biofilm mass without causing a reduction in elongated flagellar filament length (42). A previous study by Kalmokoff et al. observed that elimination of *flaG* caused a reduction in biofilm mass in another *C. jejuni* strain, and FlaG was found in the extracellular biofilm material (56). However, no other contributions of FlaG to biofilm development were identified. One possibility is that FlaG could be a component of the extracellular matrix. Treatment of established *C. jejuni* biofilms with Proteinase K severely damaged the integrity of biofilms, suggesting that proteins contribute to the biofilm matrix (64).

We previously reported that FlaG interacts with FlhA in the fT3SS export gate to antagonize FliS delivery of flagellins for secretion as a mechanism to regulate flagellar filament length (42). This interaction also results in the secretion of FlaG from the *C. jejuni* cell, which seems counterintuitive to its role in controlling the delivery of flagellins to the export gate for filament length regulation if it needs to bind FlhA to do so. It is possible that the interaction of FlaG with FlhA in the fT3SS export gate may allow FlaG to function in a mechanism to regulate filament length and also be secreted for an undefined extracellular role in biofilm formation. Further exploration will be required to determine how FlaG, and possibly its secretion, contribute to biofilm formation.

An *in vivo* biological impact of filament length was investigated by assessing the ability of WT *C. jejuni* and filament length mutants to colonize chicks. This analysis revealed that only the shortening of flagellar filaments reduced colonization throughout the intestinal tract. The most significant reductions in colonization by *C. jejuni flhA*_F485A V513G_ were observed in the upper intestinal tract. Considering that this short filament mutant showed reduced swimming velocity at low viscosity, it is possible that the viscosity conditions in the upper intestinal tract are not ideal for this mutant to colonize or persist in this region. Also, a reduction in motility may not allow the short filament mutant to acquire nutrients to support growth in this region of the intestinal tract.

In the upper intestinal tract and ceca, the elongated flagellar filament mutants colonized just as well as WT *C. jejuni*. However, we observed a trend in which both elongated and shortened flagellar filament mutants had a lower capacity to colonize the large intestines, although this reduction did not reach statistical significance. As our colonization assay only lasted for 7 days, it would be interesting to analyze whether a difference in filament length impacts long-term colonization, persistence, and transmission to other hosts. Our data may also suggest that colonization of the large intestine requires an ideal filament length to be retained in this region or prepare the bacterial cells for transmission or survival in the environment upon leaving the host.

A recurring theme from our data is that *C. jejuni* producing a shorter flagellar filament, such as the *flhA*_F485A V513G_ mutant (~1.87 µm on average), was unable to swim, aggregate, and colonize a natural host as well as WT *C. jejuni* (which produced a ~3 µm filament on average) or mutants producing longer filaments. It is curious that other polar flagellates, such as *V. cholerae* and *P. aeruginosa*, also produce flagellar filaments (~3.5–4 µm) not much longer than those of *C. jejuni* (42). However, the model peritrichous counterparts*—Salmonella* species and *E. coli*—commonly produce flagellar filaments longer than these polar flagellates and can extend up to 20 µm. A recent report has found that producing a flagellar filament 2.5 µm in length was sufficient for *Salmonella* to promote swimming motility (65). Filaments just under 4 µm could facilitate maximal swimming velocities in a low-viscosity environment. It is curious that *C. jejuni*, *V. cholerae*, and *P. aeruginosa* use orthologous FlaG proteins to restrict flagellar filament length on a population average between 3 and 4 µm, which is just above the minimum and in the optimal range for a flagellar filament to be fully functional for swimming, assuming filament dynamics for rotation and propulsion are similar between peritrichous and polar flagellates. Thus, FlaG appears to have evolved so that polar flagellates produce a filament length that meets the minimal requirement for a flagellum to function efficiently in swimming motility.

We were surprised not to observe more differences between WT *C. jejuni* and isogenic mutants producing elongated filaments. Thus, the reason why polar flagellates across bacterial species have evolved a mechanism to significantly shorten flagellar filaments relative to peritrichous bacteria has not been fully resolved. One obvious reason shorter flagellar filaments may be advantageous for some polar flagellates is in avoiding innate immunity upon infection of hosts where flagellin is an agonist recognized by TLR5 of host cells (66, 67). Polar flagellates like *Vibrio* and *Pseudomonas* species produce shortened flagellar filaments due to FlaG with flagellins that are recognized by TLR5 (68–70). In these bacteria, the flagellin epitope recognized by TLR5 is important for the structural integrity of flagellin and filament formation (67). Therefore, producing shorter filaments and less extracellular flagellin likely helps these polar flagellates by reducing recognition by TLR5 and avoiding an innate immune response. However, *C. jejuni* evades TLR5 recognition due to its flagellins lacking the TLR5 epitope (68, 71). For *C. jejuni*, glycosylation of flagellin and other alterations compensate for the requirement of the TLR5 epitope for polymerization into filaments (71). Thus, *C. jejuni* likely evolved FlaG to limit flagellar filament length for biological reasons beyond evading innate immune recognition by TLR5. Additional comparative examination of WT *C. jejuni* and elongated flagellar filament mutants in other model systems for steps in infection leading to diarrheal disease or in conditions outside the host may reveal an additional beneficial link between fine control of flagellar filament length and the fitness of *C. jejuni* cells.

## MATERIALS AND METHODS

### Bacterial growth and storage

All *C. jejuni* strains and plasmids used in this study are indicated in (Table 2) and (Table 3), respectively. All *C. jejuni* were grown under microaerobic conditions (85% N_2_, 10% CO_2_, and 5% O_2_) at 37°C in MH agar or broth with appropriate antibiotics. The following antibiotic concentrations were used when necessary: 10 µg/mL trimethoprim, 15 µg/mL chloramphenicol, 50 µg/mL kanamycin, or 0.5, 1, 2, or 5 mg/mL streptomycin. *C. jejuni* strains were stored at −80°C in 85% MH broth-15% glycerol. For most assays, strains were grown from frozen stocks for 48 h and restreaked for an additional 16 h prior to performing assays or genetic manipulations. *E. coli* strains were grown at 37°C under aerobic conditions in Luria Broth (LB) and stored at −80°C in 80% LB broth-20% glycerol. The following antibiotic concentrations were used when necessary: 100 µg/mL ampicillin, 100 µg/mL kanamycin, and 12.5 µg/mL tetracycline.

**TABLE 2:** Bacterial strains used in this study

Strain	Genotype	Source/reference
*E. coli* strains		
DH5a	*E. coli supE44* ∆*lacU169* (φ80*lacZ*DM15) *hsdR17 recA1 endA1 gyrA96 thi-1 relA1*	Invitrogen

*C. jejuni* strains		
DRH212	81-176 *rpsL*^Sm^; WT strain	72
DRH461	81-176 *rpsL*^Sm^△*astA*; WT strain	73
DRH9406	81-176 *rpsL*^Sm^△*astA* △*flaG flhA*_N436A L439A_	This study
AAW375	81-176 *rpsL*^Sm^△*astA flhA*_F485A V513G_	42
AAW417	81-176 *rpsL*^Sm^△*astA flaAB::cat-rpsL*	This study
AAW479	81-176 *rpsL*^Sm^△*astA flhA*_N436A L439A_	42
WPK469	81-176 *rpsL*^Sm^△*astA* △*flaG*	42
DAR415	81-176 *rpsL*^Sm^ *lgtF*::*cat-rpsL*	This study
DAR7653	81-176 *rpsL*^Sm^△*astA vidB*::*cat-rpsL*	This study
DAR7658	81-176 *rpsL*^Sm^△*astA* △*flaG vidB*::*cat-rpsL*	This study
AJC106	81-176 *rpsL*^Sm^△*astA flhA*_F485A V513G_ *vidB*::*cat-rpsL*	This study
AJC107	81-176 *rpsL*^Sm^△*astA flhA*_N436A L439A_ *vidB*::*cat-rpsL*	This study
ABT1266	81-176 *rpsL*^Sm^△*fliJ*	20

**TABLE 3:** Plasmids used in this study

Plasmids for construction of *C. jejuni* strains		
pUC19	Amp^R^; general cloning vector	New England Biolabs
pDRH265	Source of *cat-rpsL* cassette	72
pABT1081	pUC19 containing *flaAB*::*cat-rpsL*	20
pDAR350	pUC19 with DNA fragment containing *lgtF* with 1000 bases of upstream and downstream sequence cloned into the BamHI site	This study
pDAR367	SmaI-digested *cat-rpsL* cassette cloned into the BstBI site of *lgtF* in pDAR350	This study
pDAR6214	pUC19 containing *vidB*::*cat-rpsL*	28

### Construction of *C. jejuni* mutants

All *C. jejuni* mutants were constructed by electroporation of plasmid DNA or natural transformation with *in vitro* methylated plasmid DNA (72, 74). All plasmids containing DNA constructs to create strains and mutants for analysis were constructed by ligation of DNA fragments by T4 ligase.

Approximately 1 kb upstream and downstream of the *lgtF* locus was amplified from *C. jejuni* 81-176 genomic DNA by PCR with primers containing 5′ BamHI ends. The PCR fragment was then digested with BamHI and ligated into pUC19 to create pDAR350 (pUC19*::lgtF*). A SmaI *cat-rpsL* cassette was inserted into the BstBI site of *lgtF* in pDAR350 to create pDAR367 (72).

Plasmids pABT1081 and pDAR367 were introduced into DRH212 (81-176 *rpsL*^Sm^) or DRH461 (81-176 *rpsL*^Sm^Δ*astA*), and potential transformants were recovered on MH agar containing chloramphenicol (20, 72, 73). Mutants were verified by colony PCR to result in AAW417 (81-176 *rpsL*^Sm^Δ*astA flaAB::cat-rpsL*) and DAR415 (81-176 *rpsL*^Sm^ *lgtF::cat-rpsL*). pDAR6214 was introduced into DRH461, WPK469, AAW375, and AAW479 to replace WT *vidB* with *vidB::cat-rpsL* (28, 42, 73). Mutants were verified by colony PCR to result in DAR7653 (81-176 *rpsL*^Sm^Δ*astA vidB::cat-rpsL*), DAR7658 (81-176 *rpsL*^Sm^Δ*astA* D*flaG vidB::cat-rpsL*), AJC106 (81-176 *rpsL*^Sm^Δ*astA flhA*_F485A V513G_ *vidB::cat-rpsL*), and AJC107 (81-176 *rpsL*^Sm^Δ*astA flhA*_N436A L439A_ *vidB::cat-rpsL*).

### Measurement of the swimming velocity of individual cells

Methylcellulose (4,000 cP; Fisher) was added to MH broth immediately after autoclaving and stirred overnight to achieve a viscosity of 40 cP (28). Final viscosity was verified using a Gilmont falling ball viscometer. For measurement of swimming velocity of individual cells in MH broth alone (1 cP) or in MH broth with methylcellulose (40 cP), *C. jejuni* strains were grown, prepared for microscopy, and analyzed as previously described (28). Briefly, strains were resuspended from plates in MH broth without methylcellulose and diluted to an OD_600_ of 0.8. One milliliter of culture was added to 20 mL of MH broth, along with appropriate antibiotics, with or without methylcellulose. Cultures were incubated without shaking for 24 h at 37°C in microaerobic conditions. After growth, strains were diluted to an OD_600_ between 0.1 and 0.4 in the same viscosity of MH that they were grown. Samples of cultures were applied to microscope slides precleaned with 100% ethanol and then overlayed with a cover slip. Motility tracks of individual cells were visualized using an Olympus BX-40 microscope adapted for dark-field microscopy with a UPlanFL 100X objective and recorded with an Orca-Spark (Hamamatsu) camera and HCImage Live software. Motility tracks of individual cells were processed to calculate swimming velocity using ImageJ and an in-house Python script. Velocity measurements for at least 100 cells were examined for each strain for each experiment. Each experiment was performed three times, with a representative assay reported. Statistical analysis was calculated by ordinary one-way analysis of variance (ANOVA) followed by Tukey’s multiple comparisons post hoc test (*, *P* < 0.05).

### Autoagglutination assay

*C. jejuni* strains were grown from freezer stocks on MH agar with appropriate antibiotics for 48 h in microaerobic conditions at 37°C. Strains were restreaked on fresh MH agar and grown for an additional 16 h in microaerobic conditions at 37°C. Bacteria were resuspended from agar plates and diluted to an OD_600_ of 1.0 in 10% MH broth-90% PBS solution. Suspensions of cells were then placed into conical tubes or cuvettes and allowed to stand for up to 8 h at 25°C or 37°C. Images of standing cultures in cuvettes were captured over time. OD_600_ readings of cells in cuvettes were acquired by a spectrophotometer over time to measure clearing of the suspension as cells aggregated. All experiments were performed in triplicate. Statistical analysis was calculated by two-way ANOVA followed by Dunnett’s multiple comparisons post-hoc test.

To visualize aggregates forming in standing cultures, the assay was performed as above at 25°C, using 20 mL of each culture in a 50 mL conical tube containing a cleaned glass slide. At indicated time points, the slide was removed, and cells were visualized using an Olympus BX-40 microscope adapted for dark-field microscopy with a UPlanFL 40× objective and recorded with an Orca-Spark (Hamamatsu) camera and HCImage Live software. Images were analyzed and cropped in ImageJ software.

### Biofilm assay

Strains were grown from freezer stocks on MH agar with appropriate antibiotics for 48 h in microaerobic conditions at 37°C, then restreaked on MH agar and grown for an additional 16 h. Strains were resuspended and diluted to an OD_600_ of 0.2 in MH broth. One hundred microliter of cultures were then inoculated into 1 mL MH broth in 24-well polystyrene plates. Plates were incubated at 37°C for 3 d without shaking in microaerobic conditions. Assays were performed with three biological replicates and at least three independent times. Culture media were removed from the wells, and 500 µL of 1% crystal violet solution in 100% EtOH was added to each well and incubated at room temperature for 15 min. After incubation, the crystal violet solution was removed, and wells were washed twice with water. Plates were then allowed to dry overnight. To quantify crystal violet staining, 2 mL of solubilization buffer (80% dimethyl sulfoxide [DMSO]) was added overnight to solubilize biofilm material. One-milliliter aliquots of each strain were taken and measured at OD_570_. Statistical significance was determined using one-way ANOVA with Tukey’s multiple comparison post hoc test.

### Immunoblot analysis of proteins in whole-cell lysates and supernatants

After growth from freezer stocks, *C. jejuni* strains were restreaked on MH agar with appropriate antibiotics and grown for another 16 h in microaerobic conditions at 37°C. Strains were diluted to an OD_600_ of 0.2 in 65 mL of MH broth and then incubated at 37°C in microaerobic conditions for 20 h. Final OD_600_ measurements were taken. Culture supernatants were recovered and precipitated with trichloroacetic acid (TCA; 10% final concentration) as previously described (19, 20, 42). Precipitated proteins were then resuspended in 1 M Tris pH 9.0 buffer, and an equivalent volume of 1× SDS-PAGE loading buffer containing 5% β-mercaptoethanol was added based on the final OD_600_ measurements to normalize the amount of protein to culture density. Cellular pellets from respective cultures were resuspended in MH broth, equivalent to an OD_600_ of 0.8, based on the density of cultures after growth. For whole-cell lysates (WCLs), 1 mL aliquots were centrifuged for 3 min at 15,600 rpm in a microcentrifuge. Cell pellets were washed in 1× PBS and centrifuged again. Pellets were resuspended in 50 µL 1× SDS-PAGE loading buffer containing 5% β-mercaptoethanol.

Proteins were transferred to polyvinylidene difluoride (PVDF) membranes for immunoblotting analysis according to standard procedures. For analysis of WCLs, the following volumes were loaded in SDS-PAGE gels: FlaG, 20 µL; RpoA, 12.5 µL; FedB, 2.5 µL; CiaI, 20 µL. For detection of TCA-precipitated supernatant proteins, the following volumes were loaded onto SDS-PAGE gels: FlaG, 20 µL; RpoA, 20 µL; FedB, 10 µL; CiaI 15 µL. Primary antisera was added to membranes under the following conditions for 1–2 h or overnight: FlaG GP193 1:500 (42); RpoA GP275 1:4000 (42); FedB 1:10,000 (19); CiaI M160 1:2,000 (19). Secondary horseradish peroxidase (HRP)-conjugated antibody to detect each primary antibody was used at a concentration of 1:5,000 or 1:10,000 and applied to membranes for 1 h. Immunoblots were developed by using the Western Lightning Plus ECL Kit (Perkin-Elmer).

### Commensal colonization capacity of *C. jejuni* strains for the avian intestinal tract

The ability of WT *C. jejuni* and mutant strains to colonize the intestinal tract of chicks after oral inoculation was determined as previously described (2). Briefly, fertilized chicken eggs were incubated for 21 d at 37.8°C with appropriate humidity and rotation in a Sportsman II model 1502 incubator (Georgia Quail Farms Manufacturing Company). Strains were grown from freezer stocks on MH agar at 37°C in microaerobic conditions for 48 h and then restreaked on fresh MH agar plates and grown for another 16 h. Bacterial growth was resuspended from agar plates and diluted in 1× PBS to an OD_600_ of 0.4. Dilutions of cultures were made, and inocula were plated on MH agar with trimethoprim to determine the number of bacteria in each inoculum. Approximately 24 h post-hatch, chicks were orally infected with 100 µL of 1× PBS containing approximately 10^2^ cfu of WT *C. jejuni* or mutant strain. At day 7 post-infection, chicks were sacrificed, and contents from the proximal and distal small intestine, ceca, and large intestine were recovered and resuspended in 1× PBS to a final volume of 0.1 g organ content per mL of PBS. Serial dilutions were plated on MH agar with trimethoprim and cefoperazone and then grown for 72 h at 37°C in microaerobic conditions. Colonies were then enumerated to determine cfu per gram of content for each chick. Statistical analysis was calculated by Mann-Whitney Test. All use of animals in experimentation has been approved by the Institutional Animal Care and Use Committee at the University of Texas Southwestern Medical Center.

## SUPPLEMENTAL MATERIAL

Figure S1 jb.00199-25-s0001.pdf Immunoblot analysis of *C. jejuni proteins in whole-cell lysates and supernatants*.

ASM does not own the copyrights to Supplemental Material that may be linked to, or accessed through, an article. The authors have granted ASM a non-exclusive, world-wide license to publish the Supplemental Material files. Please contact the corresponding author directly for reuse.
