# Supplementary material for: Impact of flagellar filament length on Campylobacter jejuni for colonization and flagellar-dependent phenotypes
Source: J Bacteriol. 2025 Aug 11;207(9):e00199-25. doi: 10.1128/jb.00199-25 (PMC12445086; doi:10.1128/jb.00199-25)
Supplement: Figure S1 — Immunoblot analysis of C. jejuni proteins in whole-cell lysates and supernatants. [file jb.00199-25-s0001.pdf]

Supplemental Figure 1

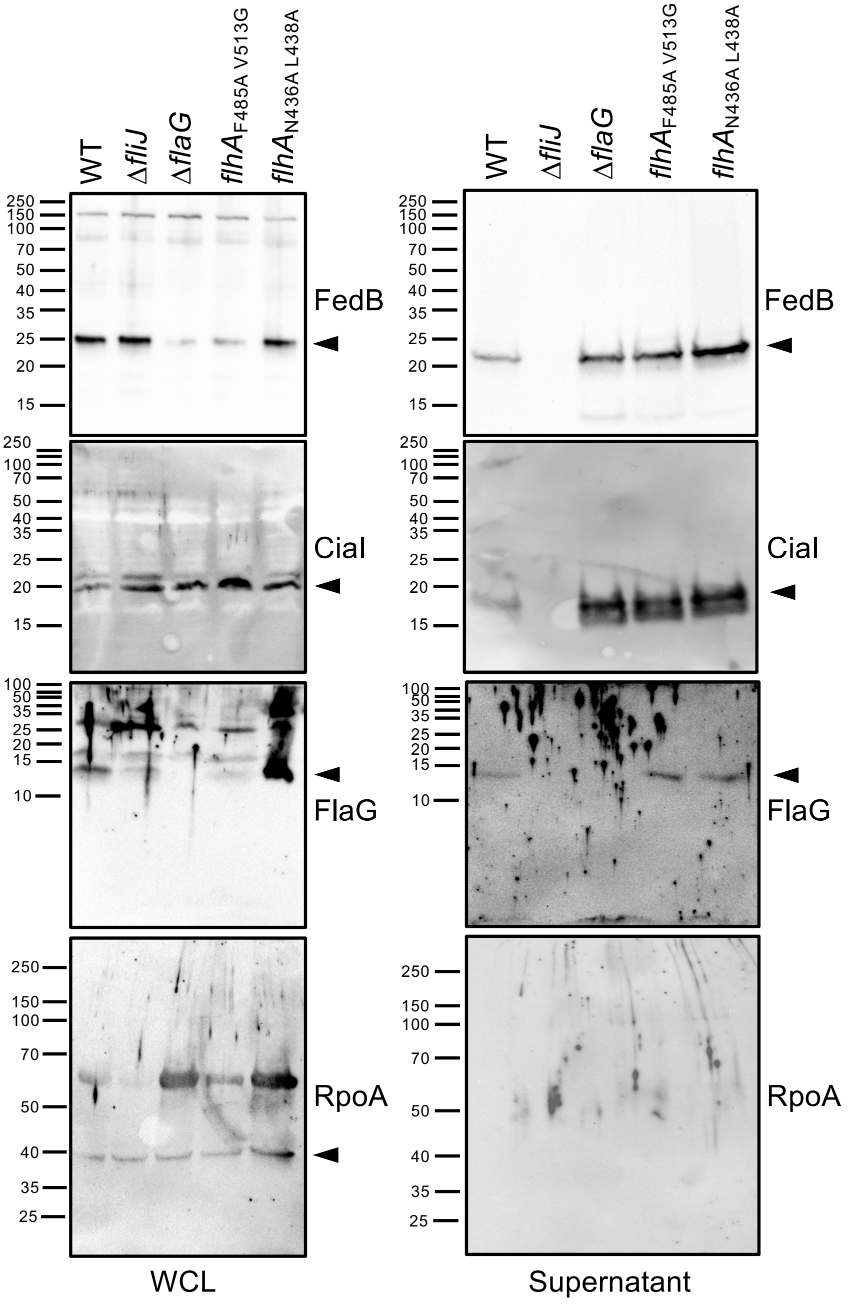

Supplemental Figure 1. Flagellar-dependent protein secretion in WT *C. jejuni* and flagellar filament length mutants. Full-size immunoblots of those in Figure 5 are shown. Arrowhead indicates the specific protein detected for analysis.
